# Sex hormone imbalance and rheumatoid arthritis in American men: a cross-sectional analysis from NHANES 2011–2016

**DOI:** 10.3389/fimmu.2024.1501257

**Published:** 2024-12-20

**Authors:** Pengfei Wen, Yidian Wang, Mingyi Yang, Xincun Qiao, Peng Yang, Shouye Hu, Lin Liu, Zhi Yang

**Affiliations:** Department of Joint Surgery, Honghui Hospital, Xi’an Jiaotong University, Shaanxi, China

**Keywords:** testosterone, SHBG, rheumatoid arthritis, NHANES, hormonal regulation, cross-sectional study

## Abstract

**Background:**

Emerging evidence suggests that sex hormones, particularly testosterone and sex hormone-binding globulin (SHBG), play a critical role in the pathophysiology of Rheumatoid arthritis (RA). However, the precise relationship between these hormonal factors and RA risk in men remains underexplored.

**Methods:**

We conducted a cross-sectional analysis using data from the National Health and Nutrition Examination Survey (NHANES) 2011-2016. A total of 3,110 male participants were included after excluding those with missing data on testosterone, SHBG, RA, or key covariates. Serum testosterone and SHBG levels were measured, and RA status was determined based on self-reported physician diagnosis. Multivariate logistic regression models were used to assess the association between testosterone, SHBG, and RA. Restricted cubic spline (RCS) regression was applied to explore nonlinear relationships. Subgroup and interaction analyses were performed to assess effect modifications by age, race/ethnicity, body mass index (BMI), hypertension, and poverty-income ratio (PIR).

**Results:**

Of the 3,110 men analyzed, 191 were diagnosed with RA. Low testosterone levels (<300 ng/dL) were significantly associated with increased RA risk (OR = 2.30, 95% CI: 1.65–3.21, p < 0.001), and elevated SHBG levels (>57 nmol/L) were also associated with a higher risk of RA (OR = 1.65, 95% CI: 1.14–2.39, p = 0.008). RCS analysis indicated a nonlinear relationship between testosterone, SHBG, and RA risk, with sharp increases in RA risk at the lower ends of testosterone and SHBG levels. Interaction analyses revealed that age, race/ethnicity, hypertension, and PIR significantly modified the relationship between these hormonal factors and RA, while BMI did not exhibit any significant interaction.

**Conclusion:**

This study provides evidence that low testosterone and high SHBG levels are associated with an increased risk of RA in men. These associations are nonlinear and modified by factors such as age, race/ethnicity, hypertension, and PIR. Our findings highlight the importance of considering hormonal status in RA risk assessment and suggest potential avenues for targeted therapeutic strategies aimed at hormonal regulation.

## 1 Introduction

Rheumatoid arthritis (RA) is a chronic autoimmune disorder characterized by synovial joint inflammation, progressive joint destruction, and potential systemic complications (1). It is more prevalent in women, but men with RA often experience more severe disease outcomes (2, 3). The etiology of RA is multifactorial, involving a combination of genetic, environmental, and hormonal factors (4, 5). Some studies have found that male RA patients have a higher risk of interstitial lung disease and cardiovascular diseases, and a higher mortality risk score, which may be related to changes in hormone levels (6, 7). In recent years, attention has turned toward the role of sex hormones, specifically testosterone and sex hormone-binding globulin (SHBG), in the pathophysiology of RA in men (8–11).

Testosterone and SHBG were chosen as the primary focus of this study due to their well-established roles in immune regulation and RA-related inflammatory pathways, particularly in men. Testosterone, a key androgen, has well-established immunomodulatory effects on various aspects of immune regulation, including cytokine production and T-cell differentiation (12, 13). Low testosterone level is linked to an increased risk of autoimmune diseases, including RA (8). Testosterone is believed to exert anti-inflammatory effects, and decreased serum testosterone levels may contribute to the exacerbation of inflammatory pathways central to RA (14). Studies suggest that men with RA often exhibit lower testosterone levels compared to healthy controls (10). Furthermore, testosterone replacement therapy has shown potential therapeutic value in some conditions (15).

Sex hormone-binding globulin (SHBG), on the other hand, plays a critical role in regulating the bioavailability of testosterone and other sex hormones (16). Elevated SHBG levels reduce the availability of free testosterone, potentially worsening RA outcomes by limiting testosterone’s anti-inflammatory effects (17). Growing evidence associates increased SHBG levels with heightened RA disease activity, suggesting that SHBG could serve as a potential biomarker for RA severity in men (11, 18). Other hormones, such as estradiol and cortisol, were not the primary focus of this study because their direct roles in male RA are less clearly defined in the literature. While estradiol has been implicated in immune modulation, its effects are primarily studied in women, and cortisol, though a stress-related hormone, lacks specificity in immune-driven RA mechanisms (19, 20).

While the association between testosterone levels and RA has been explored to some extent, the specific impact of SHBG on RA pathogenesis in men remains underexplored. Given the immunoregulatory roles of these hormones, understanding their interactions in the context of RA may provide new insights into disease mechanisms and therapeutic targets. The present study aims to investigate the relationship between serum testosterone levels, SHBG, and the risk of RA using data from the National Health and Nutrition Examination Survey (NHANES) 2011-2016. By analyzing these hormonal factors, we hope to enhance our understanding of the hormonal influences on RA pathogenesis in men and to identify possible therapeutic approaches that may improve disease outcomes.

## 2 Methods

### 2.1 Study population

**Figure 1:**
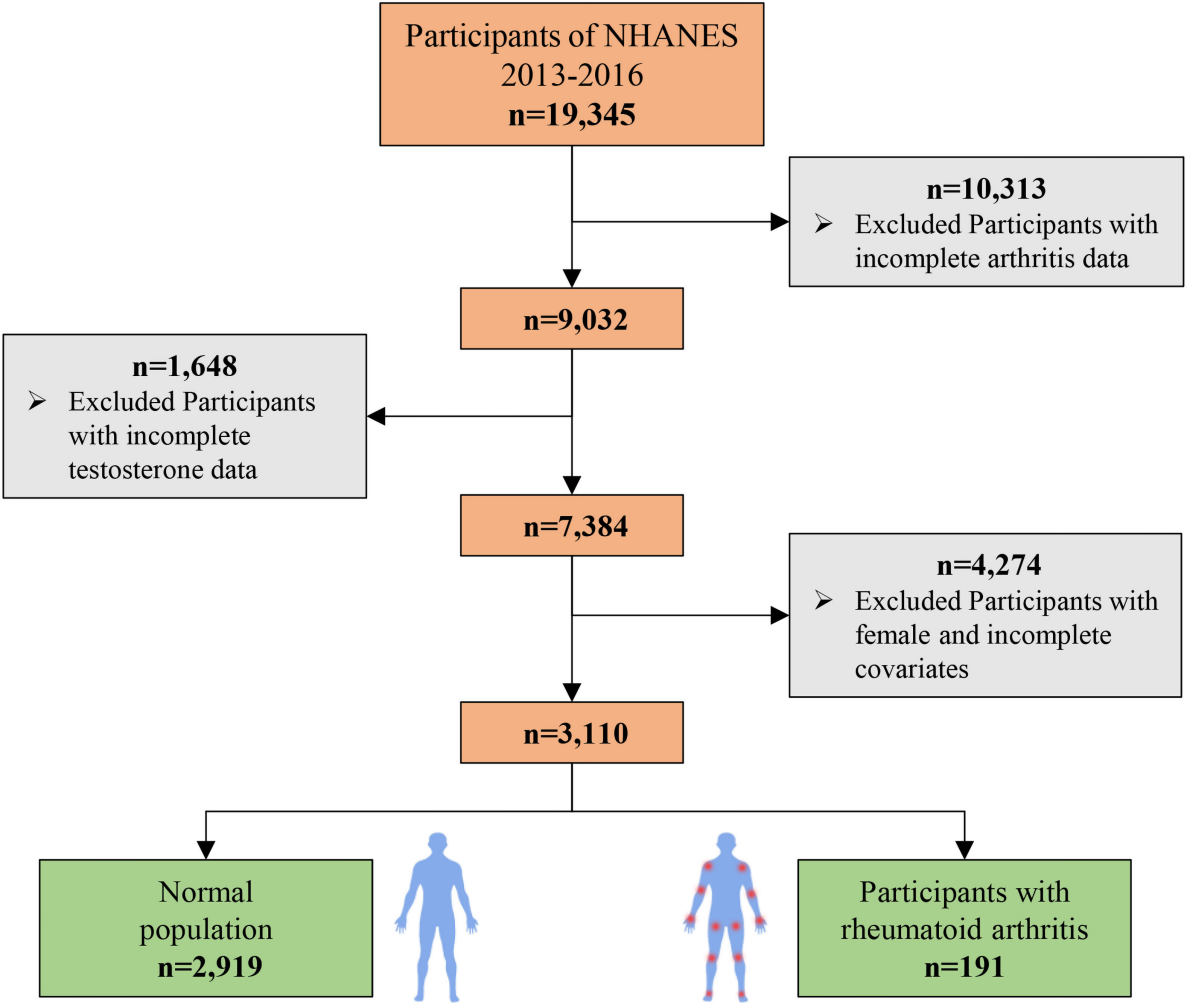
Flow chart of participant selection.

This study utilized data from the NHANES 2011–2016, which initially included 19,345 participants. The participant selection process is detailed in **Figure 1**. First, we excluded individuals with missing data on RA status, reducing the sample to 9,032 participants. Subsequently, participants with missing serum testosterone or SHBG measurements were excluded, resulting in a sample of 7,384 participants. Finally, after excluding those with missing data on covariates, the final analytic sample consisted of 3,110 participants. Among these, 2,919 individuals did not have RA, and 191 were diagnosed with RA.

### 2.2 Variables

#### 2.2.1 Testosterone

Serum testosterone levels were measured using liquid chromatography-tandem mass spectrometry (LC-MS/MS), a highly accurate method for quantifying testosterone concentrations in ng/dL. Testosterone levels were analyzed both as a continuous variable and as a categorical variable, classified into two groups: low testosterone (<300 ng/dL) and normal testosterone (≥300 ng/dL) (21, 22). The threshold of 300 ng/dL is widely recognized in clinical guidelines, including those by the Endocrine Society, as the cutoff for diagnosing hypogonadism in men (22). This level has also been used in previous studies examining testosterone’s role in autoimmune diseases, making it a biologically and clinically relevant choice for the current analysis (22).

#### 2.2.2 Sex Hormone-binding globulin

SHBG levels were measured using an electrochemiluminescent immunoassay. SHBG regulates the bioavailability of testosterone, and elevated SHBG reduces the free, biologically active testosterone in circulation. SHBG was analyzed both as a continuous variable and dichotomized into low (≤57 nmol/L) and high (>57 nmol/L) levels (23, 24). The threshold of 57 nmol/L was chosen based on population-based studies that identified this level as the upper limit of normal SHBG concentrations in men (23). Elevated SHBG levels above this threshold have been associated with an increased risk of metabolic disorders and inflammatory conditions, further supporting its relevance in the context of RA.

#### 2.2.3 Rheumatoid arthritis

RA status was determined based on self-reported physician diagnosis during the NHANES interview. Participants were asked if they had ever been told by a doctor or healthcare professional that they had rheumatoid arthritis. RA was used as a binary outcome variable (25).

#### 2.2.4 Covariates

Covariates included in the analysis were selected based on their known associations with testosterone, SHBG, and RA. These included age, race/ethnicity (Mexican American, other Hispanic, non-Hispanic Black, non-Hispanic White, other), education level (less than high school, high school graduate, some college or higher), marital status (married, widowed, divorced, separated, never married, living with partner), poverty-income ratio (PIR), smoking status, alcohol use, body mass index (BMI), hypertension, hyperlipidemia, and diabetes status (22, 23, 25). All covariates were self-reported, except for BMI, which was calculated from measured height and weight.

### 2.3 Statistical analysis

All statistical analyses were performed using SPSS version 27.0 (IBM Corp., Armonk, NY) and R version 4.4.1, accounting for the complex survey design of NHANES by applying sample weights. Descriptive statistics were used to summarize participant characteristics, with continuous variables presented as mean ± standard deviation (SD) and categorical variables as frequencies and percentages. Differences between participants with and without RA were assessed using independent t-tests for continuous variables and chi-square tests for categorical variables.

Multivariate logistic regression models were used to evaluate the association between testosterone, SHBG, and RA (26). Testosterone and SHBG were analyzed both as continuous and categorical variables. Three models were constructed: Model 1 was unadjusted, Model 2 was adjusted for age and race/ethnicity, and Model 3 was fully adjusted for education level, marital status, PIR, smoking status, alcohol use, hypertension, hyperlipidemia, diabetes, and BMI. Odds ratios (ORs) and 95% confidence intervals (CIs) were reported.

To assess the robustness of our findings, we conducted additional sensitivity analyses by restricting the analytic sample based on age and hypertension status. First, to explore whether the observed associations between testosterone, SHBG, and RA were influenced by age-related hormonal changes, we excluded participants younger than 50 years old and reanalyzed the data within the older population. Second, given the potential modifying effect of hypertension on SHBG levels and RA risk, we conducted a sensitivity analysis by excluding participants without hypertension. For both sensitivity analyses, weighted logistic regression models were used to estimate the adjusted ORs and 95% CIs for the associations of testosterone and SHBG with RA risk, consistent with the primary analysis approach. All covariates included in the primary analysis (age, race/ethnicity, BMI, etc.) were adjusted for in these models to minimize confounding.

To explore potential nonlinear associations between testosterone, SHBG, and RA, restricted cubic spline (RCS) regression was performed. RCS allows for flexible modeling of nonlinear relationships without predefining the shape of the association between continuous variables and the outcome (27). Visual representations of the spline curves were generated to examine the relationship between testosterone, SHBG, and RA risk.

Subgroup analyses were conducted to examine whether the associations between testosterone, SHBG, and RA were consistent across key covariates, including age, BMI, and smoking status. Interaction terms were tested between testosterone, SHBG, and these covariates. Forest plots were generated to present the results of the subgroup and interaction analyses. A two-sided p-value <0.05 was considered statistically significant for all analyses.

## 3 Results

### 3.1 Baseline characteristics of the study population

The baseline characteristics of the 3,110 participants included in this study are summarized in **Table 1**. The mean age of the study population was 46.70 ± 17.26 years. Among the participants, 191 individuals were diagnosed with rheumatoid arthritis (RA), while 2,919 did not have RA. Participants with RA tended to be older, with a mean age of 58.76 ± 15.15 years, compared to 45.92 ± 17.10 years in those without RA (p < 0.001).

**Table 1:** Characteristics of the study population.

Characteristics	Overall	Rheumatoid arthritis		*P*-value
		No	Yes	
n	3110	2919	191	
**Age, years**	46.70 ± 17.26	45.92 ± 17.10	58.76 ± 15.15	<0.001
Race, n (%)				0.200
Mexican American	477(15.3%)	446(14.3%)	31(1.0%)	
Other Hispanic	314(10.1%)	296(9.5%)	18(0.6%)	
Non-Hispanic Black	1191(38.3%)	1101(35.4%)	90(2.9%)	
Non-Hispanic White	621(20.0%)	580(18.6%)	41(1.3%)	
Other races	507(16.3%)	496(15.9%)	11(0.4%)	
Education, n (%)				0.626
Less than 9th grade	260(8.4%)	242(7.8%)	18(0.6%)	
9-11th grade	390(12.5%)	365(11.7%)	25(0.8%)	
High school graduate	759(24.4%)	707(22.7%)	52(1.7%)	
Some college or AA degree	880(28.3%)	819(26.3%)	61(2.0%)	
College graduate or above	821(26.4%)	786(25.3%)	35(1.1%)	
Marital Status, n (%)				0.612
Married	1710(55.0%)	1589(51.1%)	121(3.9%)	
Widowed	103(3.3%)	92(3.0%)	11(0.4%)	
Divorced	274(8.8%)	251(8.1%)	23(0.7%)	
Separated	80(2.6%)	76(2.4%)	4(0.1%)	
Never married	651(20.9%)	634(20.4%)	17(0.5%)	
Living with partner	292(9.4%)	277(8.9%)	15(0.5%)	
PIR, n (%)				0.041
<=1	628(20.2%)	587(18.9%)	41(1.3%)	
1-3	1324(42.6%)	1229(39.5%)	95(3.1%)	
>3	1158(37.2%)	1103(35.5%)	55(1.8%)	
Smoke, n (%)				0.171
Yes	1571(50.5%)	1454(46.8%)	117(3.8%)	
No	1539(49.5%)	1465(47.1%)	74(2.4%)	
Alcohol Use, n (%)				0.623
Yes	2524(81.2%)	2369(76.2%)	155(5.0%)	
No	586(18.8%)	550(17.7%)	36(1.2%)	
Hypertension, n (%)				<0.001
Yes	974(31.3%)	862(27.7%)	112(3.6%)	
No	2136(68.7%)	2057(66.1%)	79(2.5%)	
Hyperlipidemia, n (%)				0.128
Yes	1036(33.3%)	935(30.1%)	101(3.2%)	
No	2074(66.7%)	1984(63.8%)	90(2.9%)	
Diabetes, n (%)				0.735
Yes	397(12.8%)	347(11.2%)	50(1.6%)	
Borderline	73(2.3%)	62(2.0%)	11(0.4%)	
No	2639(84.9%)	2510(80.7%)	129(4.1%)	
BMI				<0.001
<25	970(31.2%)	938(30.2%)	32(1.0%)	
25-30	1226(39.4%)	1155(37.1%)	71(2.3%)	
>=30	912(29.3%)	824(26.5%)	88(2.8%)	

PIR, poverty-income ratio; BMI, body mass index.

There were no significant differences in race/ethnicity (p = 0.200), education level (p = 0.626), or marital status (p = 0.612) between the RA and non-RA groups. However, participants with RA had a significantly higher prevalence of hypertension (58.6% vs. 41.4%, p < 0.001) and tended to have higher body mass index (BMI), with 46.1% having a BMI ≥30 compared to 28.2% in the non-RA group (p < 0.001). Additionally, no significant differences were observed in smoking status (p = 0.171), alcohol use (p = 0.623), diabetes (p = 0.735), or hyperlipidemia (p = 0.128).

### 3.2 Association between testosterone, SHBG, and rheumatoid arthritis

The results of the weighted logistic regression analyses assessing the association between testosterone levels, SHBG levels, and rheumatoid arthritis are presented in **Table 2**. In the unadjusted model (Model 1), low testosterone levels (<300 ng/dL) were significantly associated with an increased risk of RA (OR = 3.30, 95% CI: 2.41–4.52, p < 0.001). This association remained significant after adjusting for age and race (Model 2: OR = 2.83, 95% CI: 2.06–3.89, p < 0.001), and in the fully adjusted model (Model 3), which controlled for marital status, education level, PIR, smoking status, alcohol use, hypertension, hyperlipidemia, diabetes, and BMI (OR = 2.30, 95% CI: 1.65–3.21, p < 0.001).

**Table 2:** Weighted logistic regression analyses of the association between testosterone and rheumatoid arthritis.

	Model 1		Model 2		Model 3	
	OR 95%CI	P value	OR 95%CI	P value	OR 95%CI	P value
Normal	Reference		Reference		Reference	
Testosterone<300	3.30(2.41-4.52)	<0.001	2.83(2.06-3.89)	<0.001	2.30(1.65-3.21)	<0.001
Normal	Reference		Reference		Reference	
SHBG>57	2.14(1.51-3.01)	<0.001	1.55(1.09-2.20)	0.015	1.65(1.14-2.39)	0.008
p for trend		<0.001		<0.001		<0.001

Model 1: Unadjusted.

Model 2: Adjusted for age and race.

Model 3: Adjusted for marital status, education, poverty-income ratio, smoking, alcohol use, hypertension, hyperlipidemia, diabetes, and BMI.

Similarly, high SHBG levels (>57 nmol/L) were significantly associated with an increased risk of RA in Model 1 (OR = 2.14, 95% CI: 1.51–3.01, p < 0.001). After adjusting for age and race (Model 2), the association weakened but remained statistically significant (OR = 1.55, 95% CI: 1.09–2.20, p = 0.015). In the fully adjusted model (Model 3), the association between elevated SHBG and RA risk remained robust (OR = 1.65, 95% CI: 1.14–2.39, p = 0.008).

A significant trend was observed for both testosterone and SHBG levels across all models (p for trend < 0.001).

### 3.3 Sensitivity analyses

In participants aged 50 years and older (**Supplementary Table 1**), sensitivity analysis revealed a significant association between low serum testosterone levels (<300 ng/dL) and an increased risk of rheumatoid arthritis (RA). In the unadjusted model (Model 1), the OR was 1.89 (95% CI: 1.29–2.75, p<0.001). After adjusting for age and race (Model 2), the OR was 1.77 (95% CI: 1.21–2.61, p=0.003). In the fully adjusted model (Model 3), the OR was 1.64 (95% CI: 1.11–2.43, p=0.013). However, sex hormone-binding globulin (SHBG) did not show a statistically significant association with RA risk after full adjustment (Model 3: OR 1.01, 95% CI: 0.67–1.53, p=0.098).

In the sensitivity analysis excluding participants without hypertension (**Supplementary Table 2**), low testosterone levels remained significantly associated with an increased risk of RA. In the unadjusted model (Model 1), the OR was 1.92 (95% CI: 1.26–2.93, p=0.002). After adjustment for age and race (Model 2), the OR was 1.69 (95% CI: 1.11–2.59, p=0.016). In the fully adjusted model (Model 3), the OR was 1.66 (95% CI: 1.07–2.58, p=0.023). Similarly, the association between SHBG and RA risk did not reach statistical significance after full adjustment (Model 3: OR 1.07, 95% CI: 0.65–1.76, p=0.278).

### 3.4 Nonlinear relationship between testosterone, SHBG, and rheumatoid arthritis

**Figure 2:**
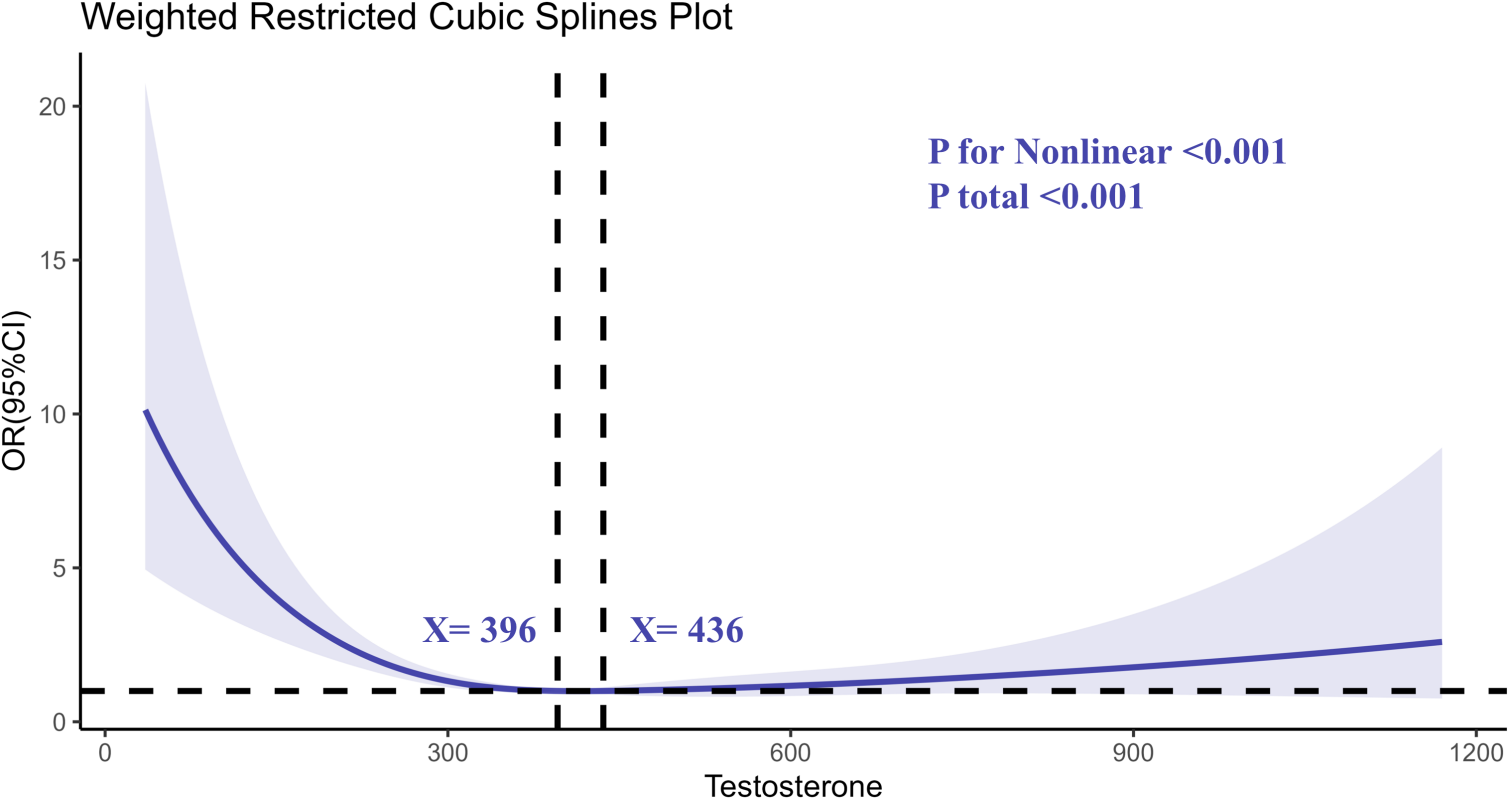
Determination of the association between testosterone and rheumatoid arthritis by restricted cubic spline (RCS) regression analysis.

Restricted cubic spline (RCS) regression analyses were conducted to explore the nonlinear relationship between testosterone, SHBG, and the risk of RA. The RCS analysis of testosterone revealed a nonlinear association between testosterone levels and RA risk (**Figure 2**). As testosterone levels decreased, the risk of RA increased sharply, particularly at levels below 300 ng/dL. The curve flattened at higher testosterone levels, indicating a stabilization in RA risk.

**Figure 3:**
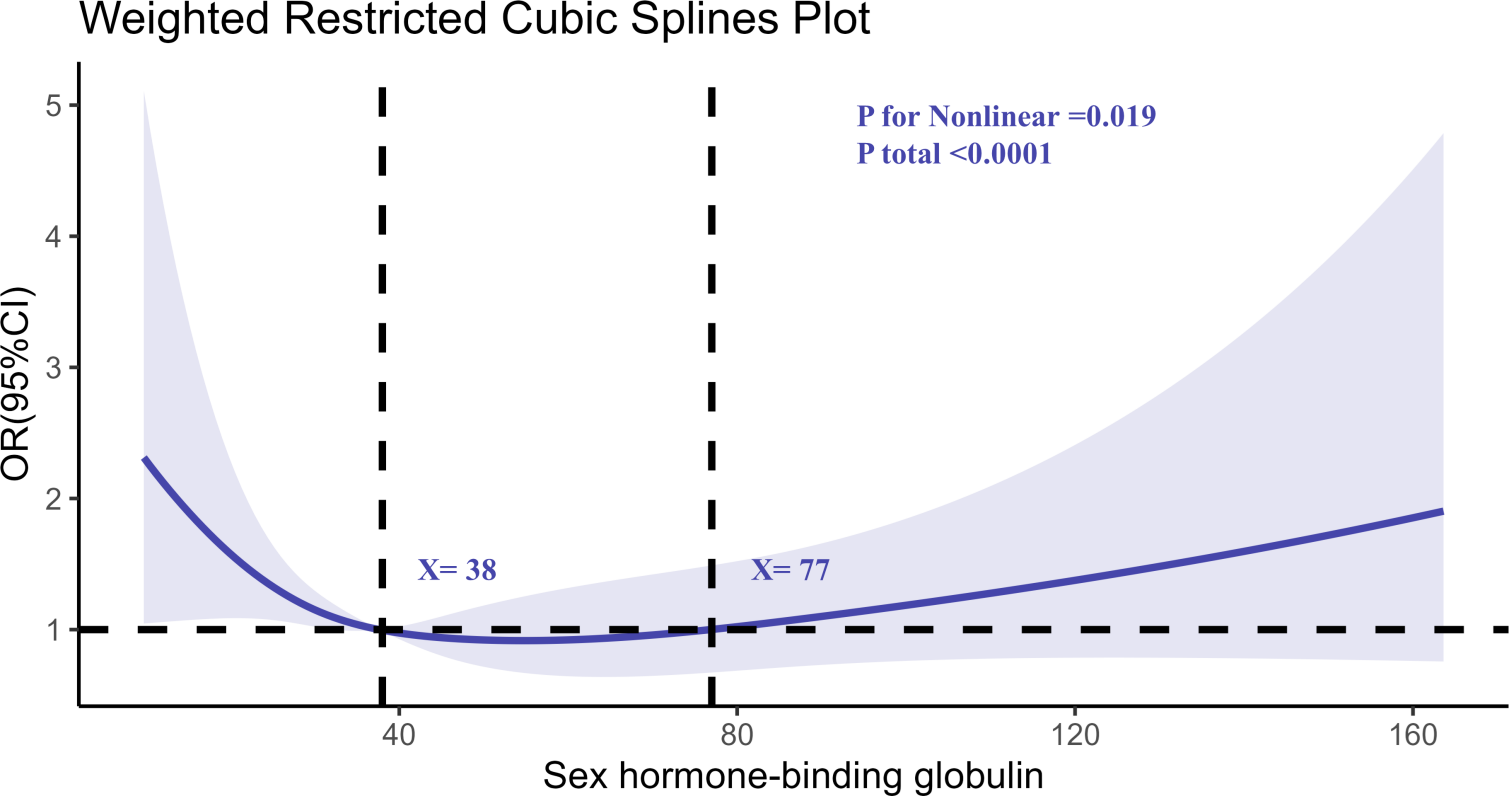
Determination of the association between SHBG and rheumatoid arthritis by restricted cubic spline (RCS) regression analysis.

Similarly, the RCS analysis for SHBG (**Figure 3**) demonstrated a nonlinear relationship. Higher SHBG levels were associated with increased RA risk, particularly at levels exceeding 57 nmol/L. The risk of RA appeared to plateau at moderate SHBG levels, with a notable increase in risk at both the low and high extremes of SHBG.

### 3.5 Subgroup and interaction analyses

**Figure 4:**
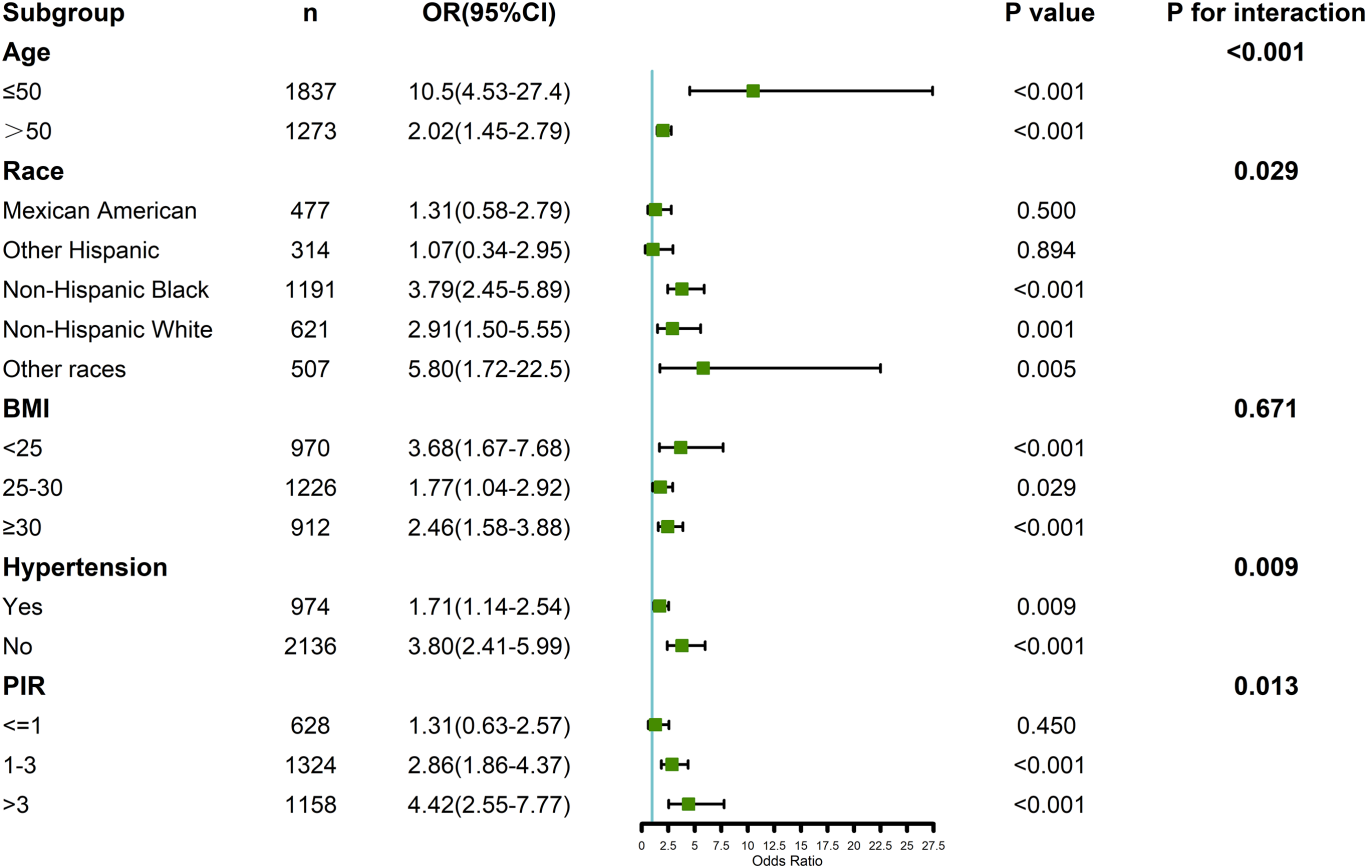
Verification of the association between testosterone, SHBG, and rheumatoid arthritis by subgroup analyses and interaction analyses.

Subgroup analyses were conducted to explore the association between testosterone, SHBG, and RA across different demographic and clinical subgroups, including age, race/ethnicity, BMI, hypertension, and PIR (**Figure 4**). Interaction analysis revealed significant interactions between testosterone, SHBG, and RA for age, race/ethnicity, hypertension, and PIR (p for interaction < 0.05). Specifically, the association between low testosterone and high SHBG levels and RA was stronger in older individuals and those with higher PIR. The additional subgroup analysis by age also yielded the same results (**Supplementary Table 3**). Furthermore, the effect of these hormones on RA risk was more pronounced in participants with hypertension and in certain racial/ethnic groups, with non-Hispanic Whites showing a stronger association compared to other groups.

However, no significant interaction was observed between BMI and the association of testosterone or SHBG with RA risk (p for interaction > 0.05), indicating that the relationship between testosterone, SHBG, and RA remained consistent across different BMI categories. Additional subgroup analyses that further controlled for BMI distribution also confirmed this result (**Supplementary Table 3**).

## 4 Discussion

In this study, we investigated the association between serum testosterone levels, SHBG, and the risk of RA among male participants using data from the NHANES 2011–2016. Our findings demonstrated that low testosterone levels and high SHBG levels are significantly associated with an increased risk of RA. Additionally, we found a nonlinear relationship between both testosterone and SHBG with RA risk, which was further influenced by factors such as age, race/ethnicity, hypertension, and PIR.

Low testosterone levels have long been suspected to play a role in the pathogenesis of autoimmune diseases, including RA (8). Testosterone is known to modulate the immune system, particularly through its effects on pro-inflammatory cytokine production and the regulation of immune cells such as T-cells and macrophages (12, 28). Our findings align with previous studies showing that low testosterone levels are associated with an increased risk of RA, potentially due to the exacerbation of inflammatory pathways (10, 29). Moreover, testosterone replacement therapy has been reported to improve symptoms in men with RA, providing further evidence of the protective role of testosterone (30–32).

Elevated SHBG levels, on the other hand, were also associated with an increased risk of RA in our study. SHBG binds to testosterone in the bloodstream, thereby reducing the availability of free, biologically active testosterone (16). Higher SHBG levels have been implicated in a variety of conditions, including cardiovascular disease and metabolic disorders (33, 34). In the context of autoimmune diseases, elevated SHBG may worsen RA outcomes by reducing free testosterone, which is known to exert anti-inflammatory effects (11, 35). Our findings add to the growing body of literature suggesting that SHBG could serve as a biomarker for RA severity, particularly in men.

The nonlinear relationship observed between testosterone and RA risk, as well as between SHBG and RA risk, is particularly noteworthy. For testosterone, we observed that the risk of RA increased sharply at lower testosterone levels, while the risk plateaued at higher testosterone levels. Similarly, SHBG exhibited a nonlinear association with RA, with increased risk observed at both low and high extremes of SHBG levels. This pattern suggests that both hormonal deficiency and excess may contribute to RA pathogenesis, a finding that is consistent with previous studies examining hormonal imbalances in autoimmune diseases (36–38). These nonlinear associations highlight the complexity of hormonal regulation in immune-mediated diseases and suggest that maintaining a balance of testosterone and SHBG may be crucial for reducing RA risk.

Our subgroup and interaction analyses further revealed that the association between testosterone, SHBG, and RA was influenced by several demographic and clinical factors. Age significantly influences the levels of serum testosterone and SHBG in men. Studies have shown that testosterone levels generally decrease with age, a condition referred to as male menopause, affecting multiple physiological systems such as muscle mass, bone density, inflammatory responses, and immune system functions (12, 13, 39). Concurrently, SHBG levels tend to rise with increasing age (39, 40), potentially impacting the availability of free testosterone and, consequently, its bioactivity (41). Our study revealed a stronger correlation between low testosterone and high SHBG with the risk of RA in older males. This implies that age-related hormonal changes might exacerbate the pathological processes of RA, particularly by affecting inflammatory responses and immune regulation (8, 9).

Moreover, the findings of our study revealed a stronger association between testosterone and SHBG levels with the risk of RA in individuals with higher PIR and among non-Hispanic whites. This indicates that socioeconomic status and racial/ethnic variations might influence RA risk by affecting hormone levels (42–44). Individuals with lower socioeconomic status may exhibit reduced testosterone and increased SHBG levels due to various stressors such as economic challenges, educational attainment, occupational stress, and living conditions (45). Nonetheless, the existing research supporting this assertion is limited, and our results do not corroborate this perspective, highlighting the intricate multifactorial nature of hormonal imbalances (46). Additionally, racial/ethnic disparities in hormone distribution may stem from differences in genetics, environmental exposures, and lifestyle choices (47). Evidence indicated that non-Hispanic whites have a higher prevalence of RA compared to other races, which is consistent with our observations (48).

Hypertension also modified the association between hormones and RA, with a stronger relationship observed in hypertensive individuals. This finding is consistent with previous studies linking hypertension and RA through shared inflammatory pathways (49, 50). Hypertension is a chronic condition characterized by systemic inflammation, oxidative stress, and endothelial dysfunction, all of which are known to influence hormone regulation (51). Decreased testosterone levels and elevated SHBG levels in hypertensive individuals may result from heightened inflammatory cytokine activity, such as C-reactive protein (CRP), interleukin-6 (IL-6), and tumor necrosis factor-alpha (TNF-α), which are implicated in both RA pathogenesis and hormonal dysregulation (52, 53). These cytokines can upregulate SHBG production in the liver, thereby reducing free testosterone levels and potentially exacerbating RA-related inflammatory responses (11). Furthermore, hypertension-induced vascular damage and metabolic dysregulation could amplify immune activation, creating a feedback loop that reinforces RA severity in individuals with low testosterone and elevated SHBG levels (12, 54). This interaction warrants further investigation in longitudinal studies to elucidate whether hypertension acts as a mediator or amplifier of the hormonal pathways influencing RA. Gaining insights into this relationship may open avenues for targeted interventions addressing both hypertensive states and hormonal imbalances in RA patients.

However, no significant interaction was observed between BMI and the associations of testosterone or SHBG with RA. This suggests that the relationship between these hormonal factors and RA is independent of BMI, contrasting with some studies that have reported an influence of obesity on hormonal regulation and autoimmune disease risk (55, 56). Additionally, other hormones such as aldosterone and androstenedione may interact with testosterone and SHBG to jointly influence the immune pathways in RA (57). Elevated aldosterone levels can promote inflammatory responses and induce vascular dysfunction, thereby indirectly affecting the levels of testosterone and SHBG (53, 58). Androstenedione, as a precursor to testosterone, may alter the availability of testosterone and consequently the binding affinity of SHBG (59); on the other hand, changes in androstenedione levels can regulate the activity of immune cells and the production of inflammatory cytokines (60). In light of the potential impacts of these hormones, we suggest that future research should analyze the relationship between the levels of these hormones and RA.

The strengths of this study include the use of a large, nationally representative dataset and the application of robust statistical techniques, such as restricted cubic spline analysis, to explore potential nonlinear relationships. However, several limitations should be acknowledged. First, the cross-sectional nature of the NHANES data limits our ability to establish causality between testosterone, SHBG, and RA risk. While we identified significant associations, the directionality of these relationships cannot be determined. For instance, it is unclear whether low testosterone and elevated SHBG levels are contributors to RA pathogenesis or consequences of preclinical or active RA. Hormonal levels may also fluctuate in response to systemic inflammation, a hallmark of RA, potentially introducing reverse causation bias. Furthermore, the lack of temporal data prevents us from assessing dynamic changes in hormonal levels over the course of disease progression. This limitation underscores the need for longitudinal studies to track testosterone and SHBG levels in relation to RA onset and severity over time. Such studies would allow for the evaluation of causal pathways and provide insights into whether interventions targeting hormonal imbalances can effectively modify disease risk or outcomes.

Second, the use of self-reported RA diagnoses may introduce misclassification bias, although self-reported data have been validated in previous population-based studies. Third, while bioavailable testosterone may provide more precise insights into testosterone’s role in RA, NHANES does not include albumin measurements necessary for its calculation. Future research incorporating bioavailable testosterone measurements is warranted to confirm and expand upon these findings. Finally, although we adjusted for a wide range of confounders, residual confounding by unmeasured factors, such as genetic predisposition, cannot be ruled out.

## 5 Conclusion

In conclusion, our study provides evidence that low testosterone and high SHBG levels are associated with an increased risk of RA in men. These associations appear to be nonlinear and are modified by factors such as age, race/ethnicity, hypertension, and PIR. Our findings contribute to the growing understanding of the hormonal influences on RA pathogenesis and highlight the importance of considering hormonal status in RA risk assessment and management. Future studies should consider designing and implementing randomized controlled trials (RCTs) to assess the efficacy of testosterone replacement therapy or SHBG-modulating treatments for patients with RA. These studies should focus on key indicators such as disease activity, joint function, and quality of life. Moreover, future hormonal intervention trials should take into account the effects of factors like age, race/ethnicity, hypertension, and PIR to achieve more personalized treatment strategies.

## Data Availability

The original contributions presented in the study are included in the article/**Supplementary Material**. Further inquiries can be directed to the corresponding authors.

## References

[1] Wen P Ma T Zhang B Hao L Wang Y Guo J. Identifying hub circadian rhythm biomarkers and immune cell infiltration in rheumatoid arthritis. Front Immunol. (2022) 13:1004883. doi: 10.3389/fimmu.2022.1004883 36238290 PMC9550876

[2] Brown P Pratt AG Hyrich KL. Therapeutic advances in rheumatoid arthritis. BMJ. (2024) 384:e070856. doi: 10.1136/bmj-2022-070856 38233032

[3] Di Matteo A Bathon JM Emery P. Rheumatoid arthritis. Lancet. (2023) 402:2019–33. doi: 10.1016/S0140-6736(23)01525-8 38240831

[4] Gravallese EM Firestein GS. Rheumatoid arthritis - common origins, divergent mechanisms. N Engl J Med. (2023) 388:529–42. doi: 10.1056/NEJMra2103726 36780677

[5] Wen P Luo P Zhang B Wang Y Hao L Wang J. Hotspots and future directions in rheumatoid arthritis-related cardiovascular disease: A scientometric and visualization study from 2001 to 2021 based on Web of Science. Front Med (Lausanne). (2022) 9:931626. doi: 10.3389/fmed.2022.931626 35966862 PMC9372309

[6] Raadsen R Hansildaar R van Kuijk AWR Nurmohamed MT. Male rheumatoid arthritis patients at substantially higher risk for cardiovascular mortality in comparison to women. Semin Arthritis Rheum. (2023) 62:152233. doi: 10.1016/j.semarthrit.2023.152233 37356211

[7] Shin S Park EH Kang EH Lee YJ Song YW Ha YJ. Sex differences in clinical characteristics and their influence on clinical outcomes in an observational cohort of patients with rheumatoid arthritis. Joint Bone Spine. (2021) 88:105124. doi: 10.1016/j.jbspin.2020.105124 33346105

[8] Panevin TS Rozhivanov RV Zotkin EG Diatroptov ME Glukhova SI Samarkina EY. Clinical and laboratory features of rheumatoid arthritis in men depending on testosterone levels. Probl Endokrinol (Mosk). (2023) 70:98–104. doi: 10.14341/probl13373 39069778 PMC11334234

[9] Raine C Giles I. What is the impact of sex hormones on the pathogenesis of rheumatoid arthritis? Front Med (Lausanne). (2022) 9:909879. doi: 10.3389/fmed.2022.909879 35935802 PMC9354962

[10] Pikwer M Giwercman A Bergstrom U Nilsson JA Jacobsson LT Turesson C. Association between testosterone levels and risk of future rheumatoid arthritis in men: a population-based case-control study. Ann Rheum Dis. (2014) 73:573–9. doi: 10.1136/annrheumdis-2012-202781 23553100

[11] Jiang Y Liu Q Alfredsson L Klareskog L Kockum I Jiang X. A genome-wide cross-trait analysis identifies genomic correlation, pleiotropic loci, and causal relationship between sex hormone-binding globulin and rheumatoid arthritis. Hum Genomics. (2023) 17:81. doi: 10.1186/s40246-023-00528-x 37644603 PMC10466838

[12] Lakshmikanth T Consiglio C Sardh F Forlin R Wang J Tan Z. Immune system adaptation during gender-affirming testosterone treatment. Nature. (2024) 633:155–64. doi: 10.1038/s41586-024-07789-z PMC11374716 39232147

[13] Ysrraelit MC Correale J. Impact of sex hormones on immune function and multiple sclerosis development. Immunology. (2019) 156:9–22. doi: 10.1111/imm.13004 30222193 PMC6283654

[14] Poole JA Thiele GM Ramler E Nelson AJ Duryee MJ Schwab AD. Combined repetitive inhalant endotoxin and collagen-induced arthritis drive inflammatory lung disease and arthritis severity in a testosterone-dependent manner. Am J Physiol Lung Cell Mol Physiol. (2024) 326:L239–51. doi: 10.1152/ajplung.00221.2023 PMC11280680 38086040

[15] Grossmann M Anawalt BD Yeap BB. Testosterone therapy in older men: clinical implications of recent landmark trials. Eur J Endocrinol. (2024) 191:R22–31. doi: 10.1093/ejendo/lvae071 38917356

[16] Jasuja R Pencina KM Lawney B Stephens-Shields AJ Ellenberg SS Snyder PJ. Modulation of circulating free testosterone fraction by testosterone, dihydrotestosterone, and estradiol during testosterone replacement therapy. Andrology. (2024) 1-8. doi: 10.1111/andr.13707 39092887

[17] Tengstrand B Carlstrom K Hafstrom I. Bioavailable testosterone in men with rheumatoid arthritis-high frequency of hypogonadism. Rheumatol (Oxford). (2002) 41:285–9. doi: 10.1093/rheumatology/41.3.285 11934965

[18] Mirone L Altomonte L D'Agostino P Zoli A Barini A Magaro M. A study of serum androgen and cortisol levels in female patients with rheumatoid arthritis. Correlation with disease activity. Clin Rheumatol. (1996) 15:15–9. doi: 10.1007/BF02231678 8929769

[19] Merrheim J Villegas J Van Wassenhove J Khansa R Berrih-Aknin S le Panse R. Estrogen, estrogen-like molecules and autoimmune diseases. Autoimmun Rev. (2020) 19:102468. doi: 10.1016/j.autrev.2020.102468 31927086

[20] Knezevic E Nenic K Milanovic V Knezevic NN. The role of cortisol in chronic stress, neurodegenerative diseases, and psychological disorders. Cells. (2023) 12:2726. doi: 10.3390/cells12232726 PMC10706127 38067154

[21] Dong X Jiang H Li S Zhang D. Low serum testosterone concentrations are associated with poor cognitive performance in older men but not women. Front Aging Neurosci. (2021) 13:712237. doi: 10.3389/fnagi.2021.712237 34790110 PMC8591394

[22] Zhan X Liu Y Chen T Wan H Xiong S Li S. The association between serum testosterone level and congestive heart failure in US male adults: data from National Health and Nutrition Examination Survey (NHANES) 2011-2016. Reprod Biol Endocrinol. (2024) 22:4. doi: 10.1186/s12958-023-01171-w 38169409 PMC10759552

[23] Yang Y Wang J Huang Y Liu Y Liu S Liu H. Association between sex hormone binding globulin and metabolic syndrome in US adults: insights from National Health and Nutrition Examination Survey (NHANES) 2013-2016. Diabetol Metab Syndr. (2024) 16:170. doi: 10.1186/s13098-024-01398-6 39026336 PMC11256583

[24] Wang Y. Definition, prevalence, and risk factors of low sex hormone-binding globulin in US adults. J Clin Endocrinol Metab. (2021) 106:e3946–56. doi: 10.1210/clinem/dgab416 PMC8571812 34125885

[25] Yin Y Dong Y Cao Y Dong G. Association of vitamin E intake with all-cause mortality among individuals with rheumatoid arthritis: A cohort study from the NHANES 1999-2018. J Am Nutr Assoc. (2024), 1–7. doi: 10.1080/27697061.2024.2401055 39259035

[26] Gu H Chen Z Zhou R Yang X Zhang Q Yang T. Vitamin D deficiency may exacerbate the role of metal exposure in depression: A cross-sectional analysis of NHANES data from 2007 to 2018. J Affect Disord. (2024) 365:265–75. doi: 10.1016/j.jad.2024.08.004 39142580

[27] Li Z Yao Z Liu Q. Association of serum calcium and metabolically healthy obese in US adults: a cross-sectional study. Ann Med. (2024) 56:2403721. doi: 10.1080/07853890.2024.2403721 39291917 PMC11411560

[28] Allahverdiyeva S Geyer CE Veth J de Vries LM de Taeye SW van Gils MJ. Testosterone and estradiol reduce inflammation of human macrophages induced by anti-SARS-CoV-2 IgG. Eur J Immunol. (2024) 54:e2451226. doi: 10.1002/eji.202451226 39246165 PMC11628899

[29] Allam JP Bunzek C Schnell L Heltzel M Weckbecker L Wilsmann-Theis D. Low serum testosterone levels in male psoriasis patients correlate with disease severity. Eur J Dermatol. (2019) 29:375–82. doi: 10.1684/ejd.2019.3605 31625919

[30] van Vollenhoven RF Houbiers JG Buttgereit F In 't Hout J Boers M Leij S. The selective estrogen receptor alpha agonist Org 37663 induces estrogenic effects but lacks antirheumatic activity: a phase IIa trial investigating efficacy and safety of Org 37663 in postmenopausal female rheumatoid arthritis patients receiving stable background methotrexate or sulfasalazine. Arthritis Rheumatol. (2010) 62:351–8. doi: 10.1002/art.27196 20112368

[31] Bove R. Autoimmune diseases and reproductive aging. Clin Immunol. (2013) 149:251–64. doi: 10.1016/j.clim.2013.02.010 PMC3805815 23522436

[32] Holroyd CR Edwards CJ. The effects of hormone replacement therapy on autoimmune disease: rheumatoid arthritis and systemic lupus erythematosus. Climacteric. (2009) 12:378–86. doi: 10.1080/13697130903025449 19591008

[33] Li MJ Jiang CQ Jin YL Zhu T Zhu F Zhang WS. Association of testosterone and sex hormone-binding globulin with all-cause and cardiovascular disease mortality in older Chinese men. J Gerontol A Biol Sci Med Sci. (2024) 79:glae065. doi: 10.1093/gerona/glae065 38394359

[34] Hedderson MM Capra A Lee C Habel LA Lee J Gold EB. Longitudinal changes in sex hormone binding globulin (SHBG) and risk of incident diabetes: the study of women's health across the nation (SWAN). Diabetes Care. (2024) 47:676–82. doi: 10.2337/dc23-1630 PMC10973900 38320264

[35] Simo R Saez-Lopez C Barbosa-Desongles A Hernandez C Selva DM. Novel insights in SHBG regulation and clinical implications. Trends Endocrinol Metab. (2015) 26:376–83. doi: 10.1016/j.tem.2015.05.001 26044465

[36] Qu Z Huang J Yang F Hong J Wang W Yan S. Sex hormone-binding globulin and arthritis: a Mendelian randomization study. Arthritis Res Ther. (2020) 22:118. doi: 10.1186/s13075-020-02202-2 32423484 PMC7236473

[37] Blanquart E Laffont S Guery JC. Sex hormone regulation of innate lymphoid cells. BioMed J. (2021) 44:144–56. doi: 10.1016/j.bj.2020.11.007 PMC8178548 33888441

[38] Lombardo G Mondelli V Worrell C Sforzini L Mariani N Nikkheslat N. Disturbed sex hormone milieu in males and females with major depressive disorder and low-grade inflammation. J Affect Disord. (2024) 356:167–76. doi: 10.1016/j.jad.2024.03.018 38494137

[39] Zirkin BR Tenover JL. Aging and declining testosterone: past, present, and hopes for the future. J Androl. (2012) 33:1111–8. doi: 10.2164/jandrol.112.017160 PMC4077344 22879528

[40] Xu P Zeng R Wan Q Xie Y Liu X An S. Aging-related increases in serum sex hormone-binding globulin levels in men might be related to increased synthesis. Exp Gerontol. (2023) 179:112249. doi: 10.1016/j.exger.2023.112249 37392803

[41] Pataky MW Young WF Nair KS. Hormonal and metabolic changes of aging and the influence of lifestyle modifications. Mayo Clin Proc. (2021) 96:788–814. doi: 10.1016/j.mayocp.2020.07.033 33673927 PMC8020896

[42] Adas M Dey M Norton S Lempp H Buch MH Cope A. What role do socioeconomic and clinical factors play in disease activity states in rheumatoid arthritis? Data from a large UK early inflammatory arthritis audit. RMD Open. (2024) 10:e004180. doi: 10.1136/rmdopen-2024-004180 PMC11253737 39004430

[43] Azizoddin DR Olmstead R Anderson KA Hirz AE Irwin MR Gholizadeh S. Socioeconomic status, reserve capacity, and depressive symptoms predict pain in Rheumatoid Arthritis: an examination of the reserve capacity model. BMC Rheumatol. (2024) 8:46. doi: 10.1186/s41927-024-00416-4 39304956 PMC11414099

[44] O'Brien J Park SH Blachley T Marchese M Middaugh N Wittstock K. Disparities in burden of disease in patients with rheumatoid arthritis across racial and ethnic groups. Clin Rheumatol. (2024) 43:921–7. doi: 10.1007/s10067-024-06869-9 PMC10876763 38267768

[45] Kutlikova HH Durdiakova JB Wagner B Vlcek M Eisenegger C Lamm C. The effects of testosterone on the physiological response to social and somatic stressors. Psychoneuroendocrinology. (2020) 117:104693. doi: 10.1016/j.psyneuen.2020.104693 32413673

[46] Harrison S Davies NM Howe LD Hughes A. Testosterone and socioeconomic position: Mendelian randomization in 306,248 men and women in UK Biobank. Sci Adv. (2021) 7:eabf8257. doi: 10.1126/sciadv.abf8257 PMC8318368 34321204

[47] Kim C Golden SH Mather KJ Laughlin GA Kong S Nan B. Racial/ethnic differences in sex hormone levels among postmenopausal women in the diabetes prevention program. J Clin Endocrinol Metab. (2012) 97:4051–60. doi: 10.1210/jc.2012-2117 PMC3485611 22879633

[48] Lane CY Lo D Thoma LM Zhang T Varma H Dalal DS. Sociocultural and economic disparities in physical therapy utilization among insured older adults with rheumatoid arthritis. J Rheumatol. (2023) 50:1414–21. doi: 10.3899/jrheum.2023-0103 37527853

[49] Ormseth MJ Oeser AM Chung CP Stein CM. Ambulatory blood pressure in patients with rheumatoid arthritis: association with immune activation. J Rheumatol. (2024) 51:870–6. doi: 10.3899/jrheum.2024-0205 PMC11368629 38749559

[50] Manavathongchai S Bian A Rho YH Oeser A Solus JF Gebretsadik T. Inflammation and hypertension in rheumatoid arthritis. J Rheumatol. (2013) 40:1806–11. doi: 10.3899/jrheum.130394 PMC3818311 23996293

[51] Moretti C Lanzolla G Moretti M Gnessi L Carmina E. Androgens and hypertension in men and women: a unifying view. Curr Hypertens Rep. (2017) 19:44. doi: 10.1007/s11906-017-0740-3 28455674

[52] Perusquia M Contreras D Herrera N. Hypotestosteronemia is an important factor for the development of hypertension: elevated blood pressure in orchidectomized conscious rats is reversed by different androgens. Endocrine. (2019) 65:416–25. doi: 10.1007/s12020-019-01978-x 31203561

[53] Jia G Guo T Liu L He C. Rheumatoid arthritis, circulating inflammatory proteins, and hypertension: A mendelian randomization study. J Clin Hypertens (Greenwich). (2024) 1-10. doi: 10.1111/jch.14932 PMC11771807 39545804

[54] Guzik TJ Nosalski R Maffia P Drummond GR. Immune and inflammatory mechanisms in hypertension. Nat Rev Cardiol. (2024) 21:396–416. doi: 10.1038/s41569-023-00964-1 38172242

[55] Kayacan Erdogan E Armagan B Kocak Ulucakoy R Orhan K Can Guven S Ozdemir Ulusoy B. Obesity might not alter tofacitinib drug survival in rheumatoid arthritis patients. Wien Klin Wochenschr. (2024). doi: 10.1007/s00508-024-02424-3 39259223

[56] Linauskas A Overvad K Symmons D Johansen MB Stengaard-Pedersen K de Thurah A. Body fat percentage, waist circumference, and obesity as risk factors for rheumatoid arthritis: A danish cohort study. Arthritis Care Res (Hoboken). (2019) 71:777–86. doi: 10.1002/acr.23694 29975015

[57] Vecchiola A Uslar T Friedrich I Aguirre J Sandoval A Carvajal CA. The role of sex hormones in aldosterone biosynthesis and their potential impact on its mineralocorticoid receptor. Cardiovasc Endocrinol Metab. (2024) 13:e0305. doi: 10.1097/XCE.0000000000000305 38846628 PMC11155591

[58] Ferreira NS Tostes RC Paradis P Schiffrin EL. Aldosterone, inflammation, immune system, and hypertension. Am J Hypertens. (2021) 34:15–27. doi: 10.1093/ajh/hpaa137 32820797 PMC7891246

[59] Simons P Valkenburg O Bons JAP Stehouwer CDA Brouwers M. The relationships of sex hormone-binding globulin, total testosterone, androstenedione and free testosterone with metabolic and reproductive features of polycystic ovary syndrome. Endocrinol Diabetes Metab. (2021) 4:e00267. doi: 10.1002/edm2.267 34277990 PMC8279613

[60] Huan C Wang M Song Y Jia Z Wei D Wang L. Inflammatory markers and androstenedione modify the effect of serum testosterone on obesity among men: Findings from a Chinese population. Andrology. (2024) 12:850–61. doi: 10.1111/andr.13544 37823215

