# Supplementary material for: Sex hormone imbalance and rheumatoid arthritis in American men: a cross-sectional analysis from NHANES 2011–2016
Source: Front Immunol. 2024 Dec 20;15:1501257. doi: 10.3389/fimmu.2024.1501257 (PMC11695343; doi:10.3389/fimmu.2024.1501257)
Supplement: Supplementary file 1 [file Table1.docx]

**Supplementary Table 1**. Sensitivity analysis: Association between testosterone, SHBG, and RA risk in participants aged 50 and above

|  | **Model 1** |  | **Model 2** |  | **Model 3** |  |
| --- | --- | --- | --- | --- | --- | --- |
|  | **OR 95%CI** | **P value** | **OR 95%CI** | **P value** | **OR 95%CI** | **P value** |
| Normal | Reference |  | Reference |  | Reference |  |
| Testosterone<300 | 1.89(1.29,2.75) | <0.001 | 1.77(1.21, 2.61) | 0.003 | 1.64(1.11,2.43) | 0.013 |
| Normal | Reference |  | Reference |  | Reference |  |
| SHBG | 1.11(1.04,1.23) | 0.047 | 0.99(0.69,1.48) | 0.055 | 1.01(0.67,1.53) | 0.098 |
| p for trend |  | <0.001 |  | <0.001 |  | <0.001 |

Model 1: Unadjusted

Model 2: Adjusted for age and race.

Model 3: Adjusted for marital status, education, poverty-income ratio, smoking, alcohol use, hypertension, hyperlipidemia, diabetes, and BMI.

**Supplementary Table 2**. Sensitivity Analysis: Association Between Testosterone, SHBG, and RA Risk in participants with hypertension.

|  | **Model 1** |  | **Model 2** |  | **Model 3** |  |
| --- | --- | --- | --- | --- | --- | --- |
|  | **OR 95%CI** | **P value** | **OR 95%CI** | **P value** | **OR 95%CI** | **P value** |
| Normal | Reference |  | Reference |  | Reference |  |
| Testosterone<300 | 1.92(1.26,2.93) | 0.002 | 1.69(1.11,2.59) | 0.016 | 1.66(1.07,2.58) | 0.023 |
| Normal | Reference |  | Reference |  | Reference |  |
| SHBG | 1.59(1.02,2.48) | 0.041 | 1.05(0.95,1.17) | 0.083 | 1.07(0.65,1.76) | 0.278 |
| p for trend |  | <0.001 |  | <0.001 |  | <0.001 |

Model 1: Unadjusted

Model 2: Adjusted for age and race.

Model 3: Adjusted for marital status, education, poverty-income ratio, smoking, alcohol use, hypertension, hyperlipidemia, diabetes, and BMI.

**Supplementary Table 3:** Subgroup and Interaction Analyses of Testosterone, SHBG, and RA Risk Stratified by Age and BMI.

| **Subgroup** | **n** | **OR(95%CI)** | **P value** | **P for interaction** |
| --- | --- | --- | --- | --- |
| **Age** |  |  |  | 0.047 |
| <40 | 1248 | 10.5(4.53,27.4) | <0.001 |  |
| 40-60 | 1020 | 1.96(1.12,3.35) | 0.016 |  |
| ≥60 | 842 | 1.95(1.29,2.94) | 0.002 |  |
| **BMI** |  |  |  | 0.893 |
| Underweight | 31 | - |  |  |
| Normal weight | 939 | 3.72(1.68,7.83) | <0.001 |  |
| Overweight | 1252 | 1.66(1.01,2.74) | 0.048 |  |
| Obesity | 888 | 2.62(1.67,4.17) | <0.001 |  |
